# Palliative care research utilising intersectionality: a scoping review

**DOI:** 10.1186/s12904-023-01310-5

**Published:** 2023-11-28

**Authors:** Helen Butler, Merryn Gott, Doctor Kate Prebble, Doctor Sarah Fortune, Doctor Jackie Robinson

**Affiliations:** 1 School of Nursing, University of Auckland, 85 Park Road, Grafton, Auckland, 1023 New Zealand; 2 School of Population Health, University of Auckland, 28 Park Road, Grafton, Auckland, 1023 New Zealand

**Keywords:** Palliative care, End-of-life care, Access to care, Healthcare disparities, Intersectionality

## Abstract

**Background:**

Access to palliative care is recognised as a human right, yet clear disparities exist. There have been recent appeals to examine people’s contexts and interactions with social systems which for many, adversely influence their utilisation of palliative care. Intersectionality provides a way to understand these drivers of inequity and ultimately advocate for change.

**Aim:**

To identify and describe published studies utilising intersectionality in relation to need, access and experience of palliative care.

**Design:**

A scoping review.

**Data sources:**

Medline, PsycINFO, CINAHL and Google Scholar databases and a manual search were undertaken for studies published up to January 2023. Included studies were evidence based articles where palliative or end of life care was the focus and intersectionality was identified and/or applied to the research that was undertaken.

**Results:**

Ten published studies were included. An analytic framework was developed to identify the extent that intersectionality was utilised in each study. A wide range of different groups were researched across the studies, with most focusing on aspects of their participant’s identity in relation to palliative care access and experience. Common topics of power, heterogeneity of people within the health system and barriers to palliative care were illuminated across the studies.

**Conclusions:**

Very limited research to date has utilised intersectionality to understand access, utilisation and experience of palliative care. This scoping review demonstrates intersectionality can provide a way to illuminate rich understandings of inequity in palliative care. It is imperative that future palliative research incorporates an intersectionality focus to further clarify the needs and experiences of structurally marginalised groups.

**Supplementary Information:**

The online version contains supplementary material available at 10.1186/s12904-023-01310-5.

## Key statements

### What is already known about the topic?

- There are clear disparities in access, utilisation and experience of palliative care for many individuals and groups based on particular social identities (age, sex, ethnicity, area of residence and socioeconomic status).
- The culture of institutions and a lack of public awareness of available palliative services can exacerbate these disparities.
- Intersectionality provides a way to examine people’s relationships with social systems and challenge discrimination and inequity.

### What this paper adds

- The paper provides the first scoping review of intersectionality in relation to need, access and experience of palliative care.
- The review finds very little literature and research informed by intersectionality has been published to date.
- The literature and research that has recognised and/or utilised intersectionality identifies people who live with multiple stigmatised identities are negatively impacted in their access, utilisation and experience of palliative care.

### Implications for practice, theory or policy

- The findings of this review have highlighted palliative research informed by intersectionality can help develop a more nuanced understanding of why some groups access and utilise palliative care and others do not.
- Further research is needed that considers and respects the lived experience of people with a focus on possible intersecting identities that may help or hinder a person to access and receive palliative care they need, when they need it.

## Background

“If you only hear one side of the story, you have no understanding at all.” Chinua Achebe.

Access to high quality palliative care is recognised as a human right [1–3]. However, clear disparities exist to palliative care access, utilisation and experience [4, 5]. Research has linked these disparities to various aspects of a person’s social identity including their age [6], sex/gender [6], race/ethnicity [7], area of residence [8] and socioeconomic status [9, 10]. Additionally, emerging evidence identifies inequalities in palliative care for people related to their social and health circumstances. For example, research undertaken in Aotearoa, New Zealand identified people with a diagnosis of mental illness are less likely to access specialist palliative care compared to the general population [11]. Similarly, inadequate palliative care provision for people who are vulnerably housed [12] or incarcerated [13] have also been identified as key equity concerns. The recent COVID-19 pandemic has further highlighted palliative care inequities experienced by differing socio-cultural groups [14]. Recent literature has challenged palliative care providers to identify ways to improve access for people by reducing structural inequalities and addressing inequity [15]. Recognising root causes of inequity, including forms of oppression will help to develop effective strategies in reducing disparities.

Intersectionality is an innovative research approach to explore health inequities and the influence of social context upon individual and group disparities [16, 17]. Intersectionality was initially conceived by Kimberlé Crenshaw to describe the exclusion of Black women from White feminist discourse and exposed the unique oppressive and discriminatory experiences they encountered within the legal justice system [18]. A core premise of intersectionality is that human lives cannot be reduced to single characteristics, or identities. Furthermore, intersectionality recognises the complexity of identity categories and contests the focus of research on normative traits rather than differences within and between identity groups [16, 19]. This is important because identifying with multiple socially unaccepted positions may compound people’s experiences of othering, discrimination and disadvantage [19–21]. However, intersectionality is more than identifying the multiple identities that people live with and how these affect health inequality on an individual level. Intersectionality invites researchers to gain a more comprehensive understanding of the dynamics that exist between individuals and systems of power such as racism, sexism, heterosexism and classism [18, 20, 22, 23]. These systems of power act to produce privilege and oppression meaning some people and groups in our society experience disparities in health, social and economic outcomes [24–26]. Intersectionality provides a way to challenge the discriminatory effects that these systems of power have on people economically, politically, culturally, subjectively and experientially [27]. Furthermore, this attention to relational aspects of power reveals people and groups at differing social locations may simultaneously experience privilege and oppression [22].

Applying intersectionality to analyse policy is one way intersectionality has already been used in palliative care to action social change. Hankivsky and colleagues developed an intersectionality based policy analysis (IPBA) framework and used this to identify people and groups who are generally not acknowledged within the palliative care policy in Canada [28]. This analysis highlighted people living in more rural and remote areas, who are socially isolated and/or stigmatised face greater barriers to accessing palliative support. This important work highlights the possibilities for intersectionality to transform palliative policy, and invites thinking of further applications of intersectionality to address inequity in palliative care. However, we are currently unclear of all causes and drivers of inequality of underserved groups in palliative care, given the limited research to date [29, 30]. It is here again that intersectionality can help. As Hancock [21] identified, intersectionality is “the best chance for an effective diagnosis and ultimately an effective prescription” (p.73). The value of intersectionality in palliative care research is starting to be recognised. There have been recent appeals in recent published literature for more palliative care research to use an intersectionality approach to understand the dynamics of inequity [31–35].

Intersectionality research can be conducted between and across various divisions in society including at individual (micro), community (meso) and national/global (macro) levels [16]. Of key focus in such multi-level analyses are the processes by which power and inequity are created, maintained and resisted in relation to a person’s intersecting identities and social location [24, 28]. In recognition of the diversity of approaches when undertaking intersectional research, Dill and colleagues [25] identified four actions to help identify intersectionality within studies. These are that the research:

1. Centres the lives of minoritised groups.
2. Understands and attends to the complexities of identity, including within-group differences.
3. Examines how systems of power manifest through structures, disciplines, cultures, and interpersonal relationships and how this impacts on people with multiple minoritised identities.
4. Includes reflection on how the research advances social change and strives toward social justice aims.

## Aim

To identify and describe published research utilising intersectionality in relation to need, access and experience of palliative care.

For the purpose of this review the following terms are defined as described in previous literature [36, 37]. Access is understood as an individual’s ability (impacted by context) to be in the position to receive healthcare. Utilisation is the actual use of the healthcare and experience includes the actual or perceived care that has occurred (including the stigma/discrimination they may have experienced).

## Method – scoping review

A scoping review was conducted to determine the extent and nature of palliative care research that had identified intersectionality. Scoping reviews are exploratory and typically focus on identifying, characterizing and summarising the evidence on a topic to identify research gaps [38–40]. They address questions such as the characteristics and contexts around concepts and phenomenon. The scoping review process includes defining a clear objective, developing a protocol, conducting the search, screening literature to ensure in meets eligibility, extracting relevant data from the included studies and writing up the findings [38, 39]. As is typical of scoping reviews, the inclusion criteria were based on relevance rather than on quality.

A search strategy was developed with support of a University of Auckland librarian. Key search terms used included palliative OR end-of-life OR palliative care OR hospice OR terminal care AND intersectionality. There was no limitation as to the date of publication. The search was conducted in Medline, PsycINFO, CINAHL and Google Scholar for all research published up until 31^st^ January 2023. A manual search was also undertaken by identifying references and research that was cited in sourced articles.

### Inclusion criteria

The inclusion criteria were:

- Published original research articles.
- Palliative or end of life care as the focus of study or discussion.
- The term intersectionality was used in the article.
- Published in the English language.

The intent of this literature review was to identify the extent and nature of the use of intersectionality within palliative research to date. Importantly, when screening for intersectionality studies, there was recognition that some authors identify their research uses intersectionality, others do not (when it is), and still others articulate their work as using intersectionality when it does not [41]. Early searches identified only a small number of results. Subsequently, considerable attempts were made to find literature that may not have explicitly identified their research or discussion as using intersectionality, but may have included commentary about intersectionality in relation to their palliative care-related topic of focus and/or findings. A hand search was also undertaken which included reviewing reference lists to capture as many relevant studies as possible. To guide decision-making on inclusion, the authors drew on the four actions outlined in the background to locate intersectionality within the literature [25].

### Exclusion criteria

Literature that may have used the term intersectionality that did not relate to access, utilisation or experience of palliative care were excluded. Literature reviews, grey literature, books, book chapters, opinion pieces and editorials were also excluded.

### Analysis

The following information was extracted from each study: design of study, country and setting of study, study samples and participant characteristics, focus area of studies, researcher’s interpretation of Intersectionality, investigation of intersectionality approach in studies and findings related to intersectionality, access and experience of palliative care. This information was collected to provide a descriptive summary of research on palliative care using intersectionality.

An intersectionality identification framework was developed by the authors to investigate the included studies in order to quantify the extent intersectionality was utilised in each study. The elements of intersectionality were drawn from the previously identified four actions and other intersectionality research and literature [41–45]. The final framework focused on eight aspects of intersectionality:

1. That the research centred the lives minoritised groups.
2. Two or more categories of identity were included in the research.
3. Researchers identified and clarified implied statements related to categories of oppression within the methodological approach of the study.
4. The study demonstrated understanding of the complexities of identity including within group difference.
5. The study recognised and incorporated aspects of power in relation to inequality (structural, disciplinary, cultural, interpersonal).
6. Intersectionality was included in all aspects of the study (including the theoretical framework, research question, design, data analysis and findings).
7. Researchers articulated the need for social change and addressed how it contributes to aims of social justice
8. Reflexive and positional activity was included within the study

The last element was included as there is consensus that being reflexive is key for intersectional researchers to understand how their own power and privilege informs their relationship with the research they are engaging in. Identifying positionality (including privileges, identities and worldviews influence) supports researchers to understand how they impact on the analysis, interpretation and explanation of their study participants’ lives [46, 47].

The Critical Appraisal Skills Programme (CASP) qualitative checklist tools was used to appraise the quality of the qualitative studies included in this review. The CASP tool was chosen as it is considered to be user-friendly, is devised for health-related research and has been endorsed by Cochrane and the World Health Organisation for use in synthesis of qualitative evidence [48]. The Appraisal tool for Cross-Sectional Studies (AXIS tool) was used to assess the quality of the one cross-sectional study that was included in this review. The AXIS tool addresses study design, reporting quality and the risk of bias in cross-sectional studies [49].

## Findings

The Preferred Reporting Items for Systematic Reviews and Meta-Analysis (PRISMA) was utilised as a guide to frame the literature search (See Supplementary Material). The initial database and manual search generated 391 titles and abstracts. After reading all of the abstracts (and full text as necessary), ten studies met the inclusion criteria. All literature that met the inclusion criteria are outlined in Table 1.

**Table 1:** Published research included in scoping review

Author and year of publication	Country	Research type	Research Design	Participants	Research Question or Aim	How researcher(s) defines and understands intersectionality
Baskaran & Hauser (2022) [50]	Nepal	Qualitative	Semi-structured interviews (*n* = 29)	29 participants including LGBQTI + palliative patients (*n* = 8), health professionals (*n* = 5), administrators (*n* = 14), community or family (*n* = 2)	Explores LGBTQI + experiences in hospice and palliative care provided by Blue Diamond Society (NGO which provides a hospice and palliative program for the LGBQTI + community)	Did not define. Identified as a theme in findings. Related intersectionality to experience of participants – intersectionality between their gender/sexuality, culture and the environment
Dworzanowski-Venter, B. (2017) [51]	South Africa	Qualitative	Interviews (*n* = 19) conducted between 2005–2013	Black male caregivers (*n* = 5, 2005 & 2010; *n* = 1 2013), female caregiver (*n* = 1 2013) and a supervisor (*n* = 1 2013) from a home-based care provider. Oncology nurses of both genders (*n* = 8 2006; *n* = 2 female 2010) from hospital setting	How can the intersection of gendered work norms and class status shape the masculine identities of black caregivers in South Africa? Does class impact on gendered work and social norms regarding feminization of care work?	Draws on previous work (Folbre, 2016) that illuminates the dynamic nature of class-gender intersectionality. Recognises the debate of using intersectionality as an analytical tool – and the care to avoid reinforcing and perpetuating oppression for certain groups. Reinforces the need for researchers to be reflexive and aware of the potential bias related to their privilege
Giesbrecht et al. (2012) [52]	Canada	Qualitative	Secondary analysis of semi-structured (*n* = 50) phone interviews	Front-line palliative care providers (*n* = 50) across the 5 provinces of Canada	To explore how frontline palliative care providers understand diversity of family caregivers and how this shapes their end-of-life caregiving experience in Canada. Consider the implications to current health and social policy aimed at supporting family caregivers	Bases analysis on previous work (Hankivsky, 2009). Sees intersectionality as an approach to consider simultaneous interactions between different aspects of social identity, and the impact of systems and processes of oppression and domination. Authors use intersectionality grounded in lived experience with the aim to pursue social justice
Giesbrecht et al. (2018) [53]	Canada	Qualitative	Secondary analysis of 30 months of ethnographic fieldnotes	People experiencing structural vulnerability (*n* = 25), their support persons (*n* = 25) and formal service providers (*n* = 69)	To explore how places of formal healthcare settings shape access to and experience of palliative care for people who are structurally vulnerable	Identifies intersectionality as a critical theory which is concerned with simultaneous interactions between aspects of social difference and identity, forms of systemic oppression at micro and macro levels. These interactions are complex and interdependent
Giesbrecht et al. (2015) [54]	Canada	Qualitative	Secondary analysis of ethnographic fieldnotes and semi structured interviews	Family caregivers (*n* = 13), care recipients (*n* = 3) and homecare nurses (*n* = 11)	What socio-environmental factors facilitate family palliative caregivers’ capacities for resilience in the home setting?	Sees intersectionality as a lens to recognise that human lives are complex cannot be reduced to singular identity categories or social locations. Identifies intersectionality as way to identify the multiple variables that impact on an issue and how they relate within specific contexts
Hutson (2016) [55]	USA	Qualitative	Secondary analysis of semi-structured interviews	People with self-acknowledged diagnosis of HIV/AIDS (*n* = 9)	To explore the health access and end of life concerns for people living with HIV/AIDS in Appalachia, USA	Did not define. Identified intersectionality in findings of a participant’s lived experience related to multiple overlapping oppressions in their life
Liu et al. (2020) [56]	USA	Qualitative	Secondary analysis using data from National Study of Caregiving (NSOC) linked with data from National Health and Aging Trends Study (NHATS)	This study used data from Round 5 (conducted in 2015) and Round 7 (conducted in 2017) of NSOC. These participants are family members and others who provided unpaid care to the NHATS participants. *N* = 1206 caregivers	To explore the differences in caregiving burdens for people with dementia across the intersectionality of race and gender	Saw intersectionality as a framework to analyse the multidimensionality of lived experience for people who are marginalised. Also recognised that intersectionality provides a perspective to examine the interconnections of power and inequality
Stajduhar et al. (2019) [57]	Canada	Qualitative	Analysis of ethnographic data (observations and interviews) collected over 30 months	People experiencing structural vulnerability (*n* = 25), their support persons (*n* = 25) and formal service providers (*n* = 69)	To explore issues and experiences of accessing palliative care for people experiencing life-limiting illness and structural vulnerabilities	Identified intersectionality as a lens of understanding to explore the complex, simultaneous and interdependent interactions between social difference and identity, forms of social oppression at micro and macro levels
Suntai et al. (2023) [58]	USA	Quantitative	Analysis of Last-month-of-life interviews conducted in the year after a person dies from 2013 and 2020 of the National Health and Aging Trends Study (NHATS)	Person who was present in the final month before an individual died, who may be a family member, friend, or other relative (*n* = 914)	To examine the intersectional impact of race and gender on care quality at the end of life	Recognised intersectionality as a theory that sees membership to 2 or more vulnerable groups increases risk of hardship across a person’s life. Recognised that a person is the sum of all of their combined identities, however systems fail to recognise the uniqueness in experience of the intersection of identities
Wilson et al. (2018) [59]	Canada	Qualitative	Three focus groups (6–9 participants in each)	People identifying as LGBQTI + (*n* = 23)	To understand the lived experience of older LGBQTI + individuals in the healthcare system in Ontario and to highlight their concerns associated with last stages of life	Did not define explicitly. Recognised that understanding the intersectionality and varying social locations for people is crucial to facilitate positive aging experiences and good end-of-life care

### Design of studies

All studies except one were qualitative and used a range of methods including interviews (*n* = 2), focus groups (*n* = 1), ethnography (*n* = 1) and secondary analysis from previously undertaken research (*n* = 5). The other study was quantitative. This study used data from a national study to undertake multivariate analyses. The CASP quality appraisal and AXIS tool results for the research included in this literature review are presented in tables (See Supplementary Material S1 and S2). All the studies clearly stated aims for their research and utilised appropriate research designs to address these aims. However, inconsistencies existed in whether the researchers articulated whether ethical issues had been attended to or if the relationship between researcher and participants had been considered. All included research was identified to have made valuable contributions to understanding palliative care in relation to people’s identity complexity.

### Countries

The studies included in this review came from a range of countries including USA (*n* = 3), Canada (*n* = 5), Nepal (*n* = 1) and South Africa (*n* = 1). Whilst eight of the ten studies are in North American countries, we recognise that the range of countries included represent diversity in the type, focus, provision and availability of palliative care and services.

### Study samples and participant characteristics

The included literature addressed different groups including Medicare beneficiaries, palliative providers, family caregivers, Black male caregivers, people who are LBGQTI + , people with a diagnosis of HIV/AIDs and, what authors described as, people experiencing ‘structural vulnerability’ [50–59]. Some researchers involved a variety of participant groups in their studies. Five of the ten studies included participants who were patients (actual or potential), six studies incorporated paid caregivers (carers or health professionals) and five studies included unpaid caregivers (family or other support). Despite the wide variety of people who were researched using an intersectional approach, common topics of power, heterogeneity of people within the health system and barriers to palliative care were illuminated across the studies.

### Focus area of studies

Most studies focused on aspects of their participant’s identity in relation to palliative care access and experience. Two studies explored the experiences of people who are LGBQTI + in relation to hospice and palliative care [50, 59]. One of these, by Baskaran and Hauser [49], focused on an NGO care provider who provides a palliative care programme for the LGBQTI + community in Nepal. The other focused on the lived experience of older LGBQTI + individuals in the healthcare system in Ontario, to highlight participant’s concerns associated with end of life [59]. Two studies explored experiences of people who are structurally vulnerable in relation to palliative care [53, 57]. One study by Stajduhar and colleagues [57], explored issues and experiences of accessing palliative care for people experiencing life-limiting illness and structural vulnerabilities. The other study focused on the settings of healthcare provision, how they shape access and experience of palliative care for people who are structurally vulnerable [57]. Hutson explored the health access and end of life concerns for people living with HIV/AIDS in Appalachia, USA [55]. Several studies focused on the intersection of gender and other identities in relation to palliative care [51, 58]. One of these examined the intersectional impact of race and gender on care quality at the end of life [58]. The other by Dworzanowski-Venter [51], focused on the intersection of gendered work norms and class status. This research explored how class and social norms shape the masculine identities of black males working in the feminized role of health caregiver in South Africa. Other studies also explored caregiving in relation to palliative care [52, 54, 56]. One investigated how frontline palliative care providers understand diversity of family caregivers and how this shapes their end-of-life caregiving experience in Canada [52]. Another study focused on socio-environmental factors, and how they facilitate family palliative caregivers’ capacities for resilience in the home setting [54]. Liu and colleagues [55] explored the differences in caregiving burdens for people with dementia across the intersectionality of race and gender.

### Interpretation of Intersectionality

See Table 1 for a full articulation of how each researcher defined and/or interpreted intersectionality in connection with their study. Researchers understood intersectionality to be either a theory [53, 58], a framework [56], a lens of understanding [54, 57], an analytical tool [51], or an approach [52]. Three of the studies did not define intersectionality explicitly [50, 55, 59] with two of these only using intersectionality within findings. Hutson recognised intersectionality related to a participant’s lived experience related to the multiple oppressions that they had experienced during their lifetime [55]. Baskaran and Hauser identified intersectionality as a theme within findings which acknowledged the lived experience for people living with multiple, simultaneously oppressive identities [50].

### Investigation of intersectionality approach in studies

How each researcher utilised intersectionality in their study has been compiled in the intersectionality identification framework (see Table 2).

**Table 2:** Investigation of included studies using the intersectionality research integration identification framework

Author & year of publication	Minoritised groups centred	Categories of identity, oppression (&/or privilege)	Methodology identifies & clarifies category implicit statements	Intersectionality in all aspects of study	Recognises identity complexity	Power analysis	Articulates social justice aims	Reflexive activity
Baskaran & Hauser (2022) [50]	People who identify as LGBQTI +	Group highly intersectional including LGBQTI + , gender, socio-economic status (SES), religion, culture, environment	Not articulated	Only identified as a theme in findings	Participants identified their various marginalised identities as simultaneously oppressive. Recognised reductionist interventions don’t validate individual & group needs related to their “complex intersectionality” (p. 7)	Not identified	Urged discrimination & inequity to be seen as symptoms of social & structural injustice. Palliative care embodies, “whole person” & hospice care. Opportunity to palliate symptoms of inequity	Not identified
Dworzanowski-Venter, B. (2017) [51]	South African black male caregivers	Class, race, professional status, gender norm, SES	To understand lived experience of black male caregivers. Recognised socially created separation in gender norms related to work	Integrated in theory framework research question, design, analysis & findings	Linked colonisation to understanding of masculinity. Recognised dominant masculinity hegemony is based on a values status of professional work & associated financial prowess. Sees conflict between abstract of masculinity & reality	Dynamic nature of intersectionality which connects structured oppression & marginalization. An increase in status of one identity does not neutralize oppression in other identities	Recognised revolutionary potential—real change only possible when intersecting oppressions are unmasked & challenged	In background & rationale. Identified all self-knowledge is shaped by contextual location. Reflexivity alone cannot ameliorate white privilege. Asks if white feminist authors be sufficiently disruptive
Giesbrecht et al. (2012) [52]	Female family caregivers	Emerged in coding & analysis. Included culture, gender, geography, life course stage, material resources	Compassionate Care Benefit (CCB) is an attempt to lessen family caregiver burden – limited uptake. Diversity is often ignored in health policy & patterns of inequity are not recognised	Part of a larger study, so not an original objective. Not included in research question (focus on diversity). Used critical diversity analysis informed by intersectionality	Conveyed women are not a homogenous group. This leads to a variety of caregiving experiences (including diversity in vulnerability & inequity). Caregiving results in gendered inequity & overlap with other social location factors	Considered simultaneous interactions between different aspects of social identity, as well as the impact of systems & processes of oppression & domination	Identified CCB is not working for some. Explored the differing social/physical locations which informed the under-utilization of the benefit. Intersectionality theory a foundation for pursuit of social justice & addressing inequity	Not articulated
Giesbrecht et al. (2018) [53]	People who experience structural vulnerability	Race/ethnicity, SES, mental health issues, disability	Accepted all people at the end-of-life experience vulnerability, this is significantly amplified for people who also experience structural vulnerability	Applied in analysis to understand how differing lived experiences shape access to care for structurally vulnerable populations	Concerned with simultaneous interactions between aspects of social difference & identity	Systemic forms of oppression (e.g., racism & ableism) interact in complex ways; includes equitable distribution of resources	To inform those in power ways of directly impacting policy, practice, & system change for equitable access to palliative care for most vulnerable population groups	Not discussed
Giesbrecht et al. (2015) [54]	Palliative family caregivers	Social networks, education, employment status, geographic location of residence, housing status, life course stage	Used case studies to show the unique lived experience of palliative family caregivers. Identified that the multiple variables connected & interacted in specific contexts as important	Secondary analysis from a larger study—not an original objective. Utilised as a lens to identify aspects of socio- environmental context	Acknowledged human lives can’t be reduced to a singular category/social location. Real lived experience is more complex. Family caregiver resilience, stress, burden & burnout is shaped by socio-environmental factors & experienced differently within this group	Explored concurrent interactions of unitary categories & social locations. The combined impacts of multiple social locations & structural processes create & perpetuate health inequities	Not explicit—provided commentary about heterogeneity (including socio-environmental aspects) for this group means nurses can enhance family caregivers’ individual capacities to be resilient & identify vulnerability	Not articulated
Hutson (2016) [55]	People diagnosed with HIV/AIDS	HIV/AIDS diagnosis, sexual orientation, location of residence, religion, ethnicity	Not articulated	Only identified in findings	Participants intersecting identities seen as sources of stigma, discrimination & oppression. Often leads to social isolation. Author found difficulty identifying stigma relating to singular identity or characteristic e.g. if related to disease/sexuality/ethnicity	Not undertaken	Recognised importance for healthcare providers to understand pervasive nature of stigma & how this impacts on advance care planning & end-of-life care	Not discussed
Liu et al. (2020) [56]	Caregivers for people diagnosed with dementia	Caregiving, gender, race, ethnicity & other socio-demographic factors	To examine the dementia caregiving burden related to multiple ascribed identities & the multidimensionality of caregiving burdens	Integrated in research question, methodology, analysis & discussion	Multidimensional burdens for dementia caregivers aren’t experienced evenly by gender or by racial/ethnic groups. Influenced by differing experiences, interpretation of stressors & coping mechanisms	Identified intersectionality provides opportunity to assess the inter-connections relevant to use of power. Did not undertake power analysis within study	General statements made about need for policy change to reflect & address current inequities	Not articulated
Stajduhar et al. (2019) [57]	People who experience structural vulnerability	Group defined as highly intersectional	Barriers to palliative care likely amplified for people who are structurally vulnerable. Positionality for structurally vulnerable changes in response to external forces (e.g. policy reform)	In definition of structural vulnerability & analysis to explore the complex, concurrent & interdependent interactions between types of social difference & identity	Whilst living in poverty, people may also experience homelessness & racism, trauma, violence, social isolation, stigma related to mental health, cognitive impairment, substance use, behavioural & mobility issues, interactions with criminal-justice system &/or disability	Explored complex, simultaneous & interdependent interaction between types of social difference, identity & forms of systemic oppression (e.g. sexism, racism, ableism) at micro & macro scales	Critical theoretical perspectives of equity & social justice utilised	Engaged in reflexivity during the observations by research staff within the field notes, which included their thoughts about the observation itself, possible influences impacting on the observation & anything of note for future observations
Suntai et al. (2023) [58]	Black American People, women	Race & gender	Considers how race & gender interact to impact on quality of end of life care	Used as theoretical framework underpinning study. Analyses aimed to see intersectional effects	Recognised identity complexity in background. Considered income, post-secondary education in analyses. Not reflected on in discussion	Not identified	Found interpersonal discrimination as a factor in findings. Considered changes in healthcare that are needed – not explicit re social justice aims	Not discussed
Wilson et al. (2018) [59]	People who identify as LGB	Age, gender identity, sexual orientation	Not articulated within methodology	Issues of intersectionality articulated in abstract & discussion	Recognised significant heterogeneity in ageing people. Age, gender identity & sexual orientation seen as non-modifiable determinants. Emphasised need for the intersections of these identities to be further understood	Not identified	Understanding intersectionality is crucial to facilitate inclusive health systems that support positive aging experiences & good end-of-life care	Not discussed

There was difference across the studies regarding the extent to which researchers integrated intersectionality. The studies by Dworzanowski-Venter [51] and Liu and colleagues [53] utilised intersectionality as a theoretical framework underpinning the study and it was integrated into the research question, design, analysis, findings and discussion. Suntai and associates [58] used also used intersectionality as a theoretical framework underpinning their quantitative study. They employed univariate, bivariate and multivariate analyses to uncover intersectional interactions between race, gender and quality end of life care. The three studies by Giesbrecht and colleagues were secondary analyses of larger data sets where intersectionality was not part of the original objective [52–54]. All applied intersectionality in the analysis phase. Stajduhar and colleagues [57] utilised intersectionality as underpinning their definition of structural vulnerability and in the analysis to explain the complex interactions between social difference and identity. Three of the ten research studies identified intersectionality only as a theme or aspect in the findings or discussion [50, 55, 59].

### Findings related to intersectionality, access and experience of palliative care

There was wide recognition across the studies that palliative care services and health systems (across and within different countries) are complex, siloed and often difficult to navigate. Services often do not easily cater to the heterogeneity of people that they aim to serve, especially when people ‘do not fit’ societies’ norms or expectations. Several studies identified that the experience of multiple intersecting stigmatised identities for people is linked with differing kinds of struggles in life and results in often diverse end of life needs and preferences [50, 53, 57]. Being different and having differing needs to the normative stereotypes of a patient can cause people to become ‘invisible’ to the health system [53, 57, 59]. Moreover, identities outside the norm may not be openly acknowledged, welcomed or advocated for by health professionals [50, 53, 55, 57, 58]. Subsequent fear of discrimination, being judged around lifestyle and associated lowered self-worth leads people to avoid seeking care [53, 55, 59]. This alongside with structural/system stigma means options for accessing and receiving palliative care for these people become limited [53, 57]. For example, two studies identified that palliative care services were often discontinued for people when they or their contexts were deemed ‘risky’ [53, 57]. Stajduhar and colleagues [52] recognised that social services often then attempted to fill the health system care provision gaps when care was denied. This often involves working outside policy and practicing covertly to ensure care needs are met.

Perceived norms of roles also influence palliative caregiving capacity and ability to receive support. Whilst women are more likely to take on the caregiver role [52, 56], societal assumptions about gender and the feminized role of caregiving do not reflect the diversity of caregivers [51, 52, 56]. Dworzanowski-Venter [51] found paid male caregivers faced social stigma for performing work most often associated with women, in contrast the status of professional insulated male oncology nurses in her study from being ‘forgotten men’ within society. The studies revealed family palliative caregivers live with identity complexity with intersections of age, gender, ethnicity, family contexts, employment status and socio-economic status [52, 54, 56]. Furthermore, a person’s intersecting identities and social location led to wide variety of caregiving experiences and its consequences, including resilience, burden and burnout, economic and health inequity [52, 54, 56]. The family caregiver role led to financial burden for many. Giesbrecht and colleagues [52] identified that women family caregivers were often ineligible for government financial support, as they were often stay at home parents or working part-time. In contrast, Liu and colleagues [56] found black male caregivers are more likely to experience greater financial burden compared to white female caregivers.

A person’s living environment and socioeconomic status were shown to impact on access and provision of palliative care. Safe and secure homes situated in urban locations increase the ability to be able to access and receive palliative care [52–54, 57]. People with lower socio-economic status who in rural areas find it more difficult to access resources that are needed to provide care for someone at home, such as equipment, medication, formal respite and home care support [52–54]. Reduced resources and access to support often results in the cessation of palliative care community services [52, 57], leaving no option for care apart from the hospital environment [52].

Power and status were strong aspects across the studies in relation to access, utilisation and experience of palliative care. Marked power differences were identified across the studies between palliative clinicians and patients [53], between clinicians and caregivers [51] and between palliative care settings and patients [53]. The professional power and status that clinicians hold was a stark contrast to the experience of powerlessness for patients who had experienced discrimination related to their intersecting identities across their lifetime [53, 59]. People experiencing overlapping forms of oppression and associated powerlessness, often experience social pressure to conform to normative society constructs of ‘patients’ in healthcare settings [55]. This in turn negatively impacts on their confidence to ask questions, disclose and receive information and ultimately have end-of-life care needs and preferences met [53, 59].

## Discussion

Intersectionality is a widely accepted theory [44, 60] and has been identified as an important focus for reducing inequities in access to palliative care [26]. However, this scoping review identified only ten research studies which have adopted an intersectionality approach to date. Researchers and theorists agree there is limited guidance to integrate intersectionality into research [57], which may be one of the reasons for the limited utilisation of intersectionality in palliative care research. The studies included in this review demonstrate there are various ways to integrate intersectionality in both qualitative and quantitative methods. Moreover, they highlight the value that intersectionality can add to understanding the effects of multiple stigmatised identities on access to and receipt of palliative care.

The findings of this review reveal the stark contrast between the aims of palliative care to provide care for all who need it and the lived experience of people that do not fit the expected identity (or attributes) of a ‘typical palliative care patient’ [4, 34, 35]. It is clear that people who have intersecting identities encounter stigma, discrimination and exclusion from much needed care when facing a life-limiting diagnosis. People ‘tainted’ by assumptions associated with their ascribed identities (including a diagnosis they may have, a role they have taken on or a situation they are in) means they may experience barriers to, or even exclusion from needed care at the end of their life. Focusing on uniform groups does not address the continued stereotyping, stigma and discrimination that people who are considered to be outside the norm experience. The egalitarian perspective of the palliative approach, that all have access to the same resources, has meant the responsibility to access and engage in care is placed on the individual. The wider characteristics of a person and contexts are not been widely recognised to act as barriers [4, 34]. This is a social injustice which needs to be addressed. Further research is needed that considers and respects the lived experience of people with a focus on possible intersecting identities that may help or hinder a person to access and receive the palliative care they need, when they need it.

### Strengths and limitations

As far as we are aware, this scoping review is the first to explore palliative care published research that has used an intersectionality approach. A strength of this review is the differing foci of research undertaken as well as the variety of people and groups studied using an intersectional approach. The qualitative design utilised in most of the studies enabled deep exploration and gathering of rich information of lived experience. Despite the variety of participants and focus of studies, common themes across the research were identified. These included the diversity of people within the health system, power differences between healthcare providers and recipients of care and barriers to palliative care.

There are limitations to this review which relate to the extent and quality of the existing evidence base. Only ten published studies were included, nine of which were qualitative and one study was quantitative.. Whilst intersectionality as a frame of thinking is not new, the utilisation as a research approach means no research was undertaken prior to 2012 with respect to palliative care. It is clear that more research using an intersectionality approach is needed when exploring palliative care and particularly inequitable access and reception of care.

Findings of this literature review should be interpreted cautiously, in view of the methodological and contextual heterogeneity of included studies, which posed some challenges for analysis, summarising findings and discussion.

### Supplementary Information

**Additional file 1:** **Appendix 1:** PRISMA flow diagram

** Additional file 2:** **Supplementary Table S1.** CASP quality appraisal results for qualitative research included in literature review. **Supplementary Table S2.** AXIS quality appraisal results for quantitative research included in literature review.

## Data Availability

The authors confirm that the data supporting the findings of this study are available within the article and its supplementary materials.
