# Supplementary material for: Palliative care research utilising intersectionality: a scoping review
Source: BMC Palliat Care. 2023 Nov 28;22:189. doi: 10.1186/s12904-023-01310-5 (PMC10683236; doi:10.1186/s12904-023-01310-5)
Supplement: Supplementary file 1 — Additional file 1: Appendix 1: PRISMA flow diagram [file 12904_2023_1310_MOESM1_ESM.doc]

Appendix 1: PRISMA Flow Diagram

**Screening**

**Included**

**Eligibility**

**Identification**

Records identified through database searching
(n = 44 )

Additional records identified through other sources
(n = 16 )

Records after duplicates removed
(n = 146 )

Title and abstract screened
(n = 146 )

Records excluded
(n = 113)

Full-text articles assessed for eligibility
(n = 33)

Full-text articles excluded, with reasons
(n = 23 )

not original research (n=8)

intersectionality not discussed (n=6)

palliative care not of focus (n=8)

out of scope (n=1)

Studies included in descriptive synthesis and analysis
(n = 10 )
