# Supplementary material for: Palliative care research utilising intersectionality: a scoping review
Source: BMC Palliat Care. 2023 Nov 28;22:189. doi: 10.1186/s12904-023-01310-5 (PMC10683236; doi:10.1186/s12904-023-01310-5)
Supplement: Supplementary file 2 — Additional file 2: Supplementary Table S1. CASP quality appraisal results for qualitative research included in literature review. Supplementary Table S2. AXIS quality appraisal results for quantitative research included in literature review. [file 12904_2023_1310_MOESM2_ESM.docx]

**Supplementary Material**

Supplementary Table S1: CASP quality appraisal results for qualitative research included in literature review

| Author and year of publication | Was there a clear statement of the aims of the research? | Is a qualitative methodology appropriate? | Was the research design appropriate to address the aims of the research? | Was the recruitment strategy appropriate to the aims of the research? | Was the data collected in a way that addressed the research issue? | Has the relationship between researcher and participants been adequately considered? | Have ethical issues been taken into consideration? | Was the data analysis sufficiently rigorous? | Is there a clear statement of findings? | How valuable is the research? |
| --- | --- | --- | --- | --- | --- | --- | --- | --- | --- | --- |
| Baskaran & Hauser (2022) (50) | Yes | Yes | Yes | Yes | Yes | Can’t tell | Somewhat | Yes | Yes | Identified themes inherent to the experiences of LGBTQI+ Nepali people receiving palliative and hospice care. |
| Dworzanowski-Venter (2017) (51) | Yes | Yes | Yes | Can’t tell | Somewhat | Yes | Can’t tell | No | Somewhat | Highlights the intersection of class and gendered work norms for black male caregivers in in post-colonial South Africa. |
| Giesbrecht et al. (2012) (52) | Yes | Yes | Yes | Yes – not primary aim as secondary analysis | Yes – not primary aim as secondary analysis | Can’t tell | Yes | Yes | Yes | Revealed experiences of caregiving are not homogenous and access to services and supports are not universal across Canada. |
| Giesbrecht et al. (2018) (53) | Yes | Yes | Yes | Yes – not primary aim as secondary analysis | Yes – not primary aim as secondary analysis | Can’t tell | Yes | Yes | Yes | Highlighted the power differential and inequities in care people who are structurally vulnerable experience when accessing public healthcare settings in Canada. |
| Giesbrecht et al. (2015) (54) | Yes | Yes | Yes | Yes – not primary aim as secondary analysis | Yes – not primary aim as secondary analysis | Can’t tell | Yes | Yes | Yes | Identified the intersecting socio-environmental factors that contribute to palliative family caregiver resilience in the homecare context in Canada. |
| Hutson (2016) (55) | Yes | Yes | Yes | Yes – not primary aim as secondary analysis | Yes – not primary aim as secondary analysis | Can’t tell | Somewhat | Yes | Yes | Findings reveal the importance of culture when addressing the source of HIV stigma in the southeastern United States. |
| Liu et al. (2020) (56) | Yes | Yes | Yes | Yes – not primary aim as secondary analysis | Yes – not primary aim as secondary analysis | Can’t tell | Can’t tell | Yes | Yes | Highlighted that Black male dementia caregivers experience financial burden and White female dementia caregivers experience emotional burden disproportionately. |
| Stajduhar et al. (2019) (57) | Yes | Yes | Yes | Yes | Yes | Yes | Yes | Yes | Yes | Contributed deep insights of the needs of and barriers experienced by people who are structurally vulnerable and facing a life-limiting illness. |
| Wilson et al. (2018) (59) | Yes | Yes | Yes | Yes | Yes | Yes | Yes | Yes | Yes | Identified gender identity and  sexual orientation in relation to social connections, expectations of care, and the unique fear related to maintaining  authentic identity throughout aging and end-of-life. |

Supplementary Table S2: AXIS quality appraisal results for quantitative research included in literature review

|  | Suntai et al. (2023) (58) |
| --- | --- |
| **Introduction** |  |
| Were the aims/objectives of the study clear? | Yes |
| **Methods** |  |
| Was the study design appropriate for the stated aims? | Yes |
| Was the sample size justified? | Final sample included 914 black and white decedents who died between 2013 and 2020 who had complete data on all variables. Not articulated how many in the original dataset. Unsure how representative. |
| Was the target/reference population clearly defined? | Yes |
| Was the sample frame taken from an appropriate population base so that it closely represented the target/reference population under investigation? | Yes |
| Was the selection process likely to select subjects/participants that were representative of the target/reference population under investigation? | Yes |
| Were measures undertaken to address and categorise non-responders? | Not articulated |
| Were the risk factor and outcome variables measured appropriate to the aims of the study? | Yes |
| Were the risk factor and outcome variables measured correctly using instruments/measurements that had been trialed, piloted or published previously? | Yes |
| Is it clear what was used to determined statistical significance and/or precision estimates? (e.g. p-values, confidence intervals) | Yes |
| Were the methods (including statistical methods) sufficiently described to enable them to be repeated? | Yes |
| **Results** |  |
| Were the basic data adequately described? | Yes |
| Does the response rate raise concerns about non-response bias? | Unsure as to how many decedents did not have complete data and were therefore excluded. |
| If appropriate, was information about non-responders described? | Not articulated. |
| Were the results internally consistent? | Yes |
| Were the results presented for all the analyses described in the methods? | Yes |
| **Discussion** |  |
| Were the authors' discussions and conclusions justified by the results? | Yes |
| Were the limitations of the study discussed? | Yes several limitations identified – this was a secondary data analysis, only included black and white decedents, participants were proxies of people who died, |
| **Other** |  |
| Were there any funding sources or conflicts of interest that may affect the authors’ interpretation of the results? | No |
| Was ethical approval or consent of participants attained? | Not articulated |
